# Case report: Spinal cord and vertebral body infarction in a patient with thoracic aortic dissection

**DOI:** 10.4103/0971-3026.40297

**Published:** 2008-05

**Authors:** KS Murugan, KR Kalpana

**Affiliations:** Clarity Imaging Centre, Coimbatore - 641 009, Tamil Nadu, India

Spinal cord infarction remains a diagnostic challenge for physicians and radiologists alike, since specific clinical and imaging features are often lacking. However MRI is able to serve as a diagnostic tool, mainly because of its ability to rule out other causes of acute neurological deficit. We present a case with typical MRI imaging features of spinal cord infarction. Associated vertebral body involvement was also noted.

## Case History

A 58-year-old man came with a history of sudden onset of paraplegia 12 days back, after an episode of severe chest and abdominal pain that radiated to the back. There was no history of fever or trauma. On examination, he had flaccid paralysis of both lower limbs, with loss of bowel and bladder control. The deep tendon reflexes were absent and there was no Babinski's response. Clinically, the paraplegia was localized to the T5 level and was presumed to be of vascular origin.

**Figure 1:**
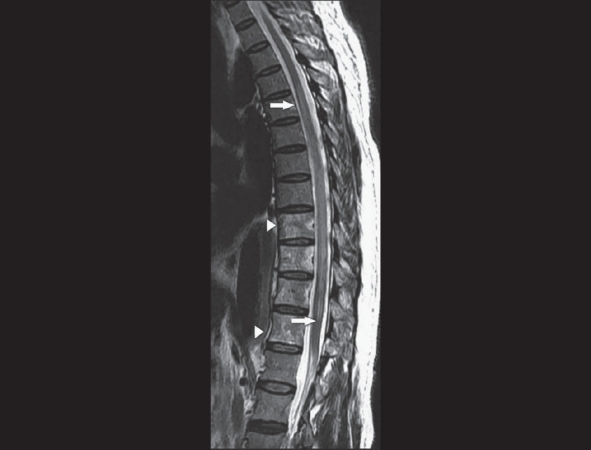
Sagittal T2W image shows cord swelling and central cord hyperintensity (arrows).Vertebral marrow hyperintensity is also noted (arrowheads)

**Figure 2:**
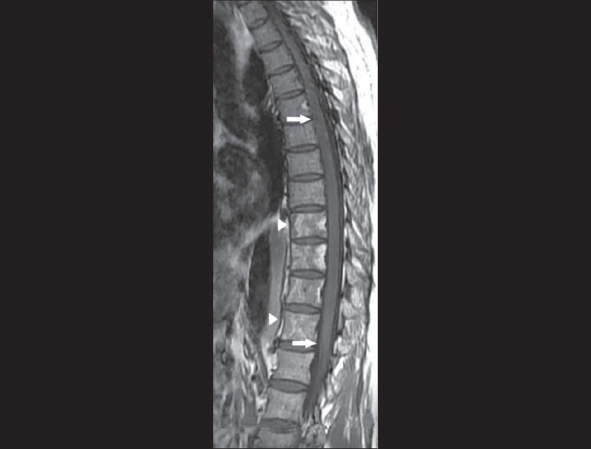
Sagittal T1W image shows only cord swelling (arrows). Vertebral marrow hyperintensity is also noted (arrowheads)

**Figure 3:**
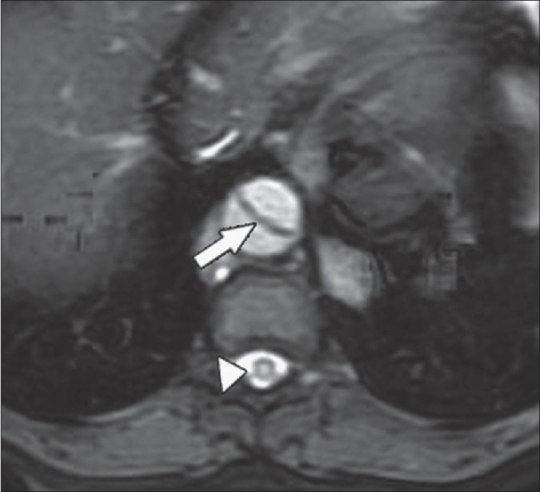
Axial T2W image shows an intimal flap in the descending aorta (arrow) and central cord hyperintensity (arrowhead)

**Figure 4:**
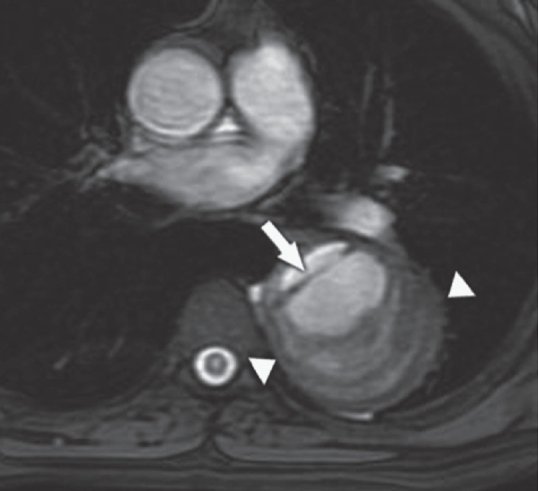
Axial FIESTA image shows an intimal flap (arrow) and a thrombosed aneurysm (arrowheads)

MRI of the thoracolumbar spine revealed focal cord enlargement from T5 to T11, with associated hyperintensity in the cord on T2W images. Altered vertebral marrow signal changes were also noted from T9 to T12 [Figures 1 and 2]. The descending thoracic aorta revealed a dissection flap from just distal to the origin of the left subclavian artery to the suprarenal segment of the abdominal aorta [Figure 3]. A thrombosed saccular aneurysmal sac was also seen in the descending thoracic aorta [Figure 4].

## Discussion

Although frequently devastating, spinal cord ischemia is an uncommon cause of myelopathy and often remains undiagnosed as acute myelopathy of unknown origin, unlike the more easily diagnosed cerebral infarcts.[1] Some studies estimate its incidence to be around 1-2% of all cases of stroke.[2] Though plain radiographs, CT, and MRI help in the accurate identification of extramedullary or intramedullary etiologies, diagnosis of spinal cord ischemia is missed in up to 59% of cases at the time of presentation.[3] Spinal cord ischemia can occur as a result of spontaneous/traumatic aortic dissection, thoracoabdominal surgery, spinal angiography, decompression sickness, vasculitis, sickle cell anemia, and hypercoagulable states.[4] Spontaneous aortic dissection is the most common cause and is responsible for about 2% of cases.

The spinal cord receives its blood supply from the longitudinal anterior spinal artery (ASA) and the paired posterior spinal arteries (PSA).The anterior medullary arteries, which are 6 to 10 in number, arise from the segmental arteries of the aorta on both sides. They reinforce the blood supply of the ASA. The thoracolumbar region, extending from the T8 segment to the conus medullaris, has a relatively rich blood supply, usually originating from a single, large radiculomedullary artery. This vessel, described by Adamkiewicz as the ‘arteria radicularis anterior magna,’ is also known as the artery of Adamkiewicz. The anterior central branches, which are nutrient arteries, arise from the segmental arteries. They supply the ventral and lateral parts of the vertebral body.

MRI findings in spinal cord ischemia are independent of the etiology of the infarction. T1W images may be normal or may show a bulky cord. Areas of intramedullary cord signal abnormality are seen best on T2W images. Signal changes often involve only the central gray matter structures, though in more severely affected patients, they are present throughout the entire cross section of the cord.[5] Enhancement of gray matter may be seen on postcontrast images.[6] Similar MRI findings may also be seen in transverse myelitis, demyelination, intrinsic cord tumors, and inflammation, but the abrupt onset of clinical symptoms and the predilection for the central gray matter are findings that help in arriving at a diagnosis of spinal cord ischemia. T2W images may also show the associated vertebral body changes. Vertebral body involvement is a rare occurrence but, when present, adds specificity to the diagnosis of spinal cord infarction in equivocal cases.[7,8] Vertebral body infarction accompanies spinal cord infarction only if the arterial occlusion is proximal to the vessels supplying the vertebral body.[7]

In the case described by us, there were central cord signal changes from T5 to T11, suggestive of infarction. This corresponds to the distribution territory of the artery of Adamkiewicz. In addition, there were vertebral body signal changes from T9 to T12, adding to the diagnostic confidence of infarction. Also the presence of aortic dissection in this case helped in clinching the diagnosis. The medullary artery has an ascending course along with the nerve roots, the obliquity of which increases from the cranial to the caudal levels. This is responsible for the segmental difference between the vertebral body and the spinal cord lesions.[9] Although the time delay for onset of vertebral body infarction is not known, the presence of indicative vertebral signal changes is highly suggestive of spinal cord ischemia if there is an evolution of these signal abnormalities (i.e., normal to abnormal) at the appropriate level.[7] Therefore, in equivocal cases of spinal cord infarction, it may be useful to do a follow-up MRI as well.
